# The Feeding Behaviour of Gall Midge Larvae and Its Implications for Biocontrol of the Giant Reed: Insights from Stable Isotope Analysis

**DOI:** 10.3390/biology11121805

**Published:** 2022-12-12

**Authors:** Giulio Careddu, Marcovalerio Botti, Massimo Cristofaro, Simona Sporta Caputi, Edoardo Calizza, Loreto Rossi, Maria Letizia Costantini

**Affiliations:** 1 Department of Environmental Biology, Sapienza University of Rome, 00185 Rome, Italy; 2 CoNISMa, National Inter-University Consortium for Marine Sciences, 00196 Rome, Italy; 3 Biotechnology and Biological Control Agency (BBCA), 00123 Rome, Italy

**Keywords:** invasive species, giant reed, gall midge, biological control, stable isotope analysis, trophic relationships

## Abstract

**Simple Summary:**

We used stable isotope analysis of carbon and nitrogen to investigate the feeding relationships between the giant reed *Arundo donax*, larvae of the gall midge *Lasioptera donacis* and the saprophytic fungus *Arthrinium arundinis*, which grows in the reed’s internodes. We also evaluated the effects of the parasitic nematode *Tripius gyraloura* on midge larval feeding behaviour. The giant reed is one of the most invasive plant species in the world and the gall midge is a potential candidate for its biological control. It is currently unknown whether the larvae can feed directly on the reed or only the fungus growing in the reeds. Furthermore, it is not clear whether midge larvae infected by the parasitic nematode have different feeding behaviour. Our results indicated that the larvae feed on both the reed and the fungus in variable proportions depending on reed quality. We also observed that parasitised and non-parasitised larvae have the same diet, indicating that infection by nematodes does not change the midge larval trophic preferences.

**Abstract:**

The gall midge *Lasioptera donacis*, whose larval stage interferes with the reed’s leaf development, is a potential candidate agent for the biological control of *Arundo donax*. Reed infestation is always associated with the presence of a saprophytic fungus, *Arthrinium arundinis*, which is believed to provide food for the larvae. Larvae also interact with a parasitic nematode, *Tripius gyraloura,* which can be considered its natural enemy. To deepen our knowledge of the plant–fungus–insect trophic interactions and to understand the effects of the nematode on midge larval feeding behaviour, we applied stable isotope analysis, one of the most effective methods for investigating animal feeding preferences in various contexts. The results showed that on average the fungus accounted for 65% of the diet of the midge larvae, which however consumed the reed and the fungus in variable proportions depending on reed quality (expressed as the C:N ratio). No differences in feeding behaviour were observed between parasitised and non-parasitised midge larvae, indicating that nematodes have no effect in this regard. Due to its trophic habits, *L. donacis* could be an effective control agent of *A. donax* and these results need to be considered when implementing biological control measures.

## 1. Introduction

Biological invasions are a significant driver of human-induced global change, being considered the second biggest cause of biodiversity loss after habitat destruction [1,2]. About 80% of endangered species around the world are currently threatened by stressors arising from invasive species, many of which were deliberately introduced for various purposes including food or timber production, landscape restoration, erosion control, aesthetics and recreation [3,4]. Interactions between invasive and native species have important implications for the structure and functioning of ecosystems [4,5,6], and their effects can be manifested in various ways, such as reduction in the richness and abundance of native species, genetic changes in native populations via hybridisation and disruption of mutualistic networks [6,7,8].

Invasive alien plants tend to establish themselves easily, expanding quickly in the new habitat [4]. Facilitated by anthropogenic activities, they tend to surmount geographical and environmental barriers [4]. They have various impacts on native plants and can have cascading effects on entire food webs [9,10,11]. In many cases these plants build dense populations that can exclude other species [12,13].

The giant reed *Arundo donax* L. (Poaceae: Cyperales) is an extremely invasive weed typical of riparian habitats, drainage ditches and irrigation channels, which has been included among the 100 most invasive species on the planet [14]. This reed is native to the Old World, spreading from the Iberian Peninsula in Europe across the Mediterranean to South Asia. It was introduced into North America in the early 1500s by colonists, where it quickly became naturalised, and this species now extends to all continents except Antarctica [15,16]. *Arundo donax* forms dense monospecific beds, becoming the dominant species in invaded riparian habitats, where it competes with native plants for water resources and reduces both plant and macroinvertebrate diversity [15,16,17,18]. Mechanical removal of *A. donax* can be extremely expensive and labour-intensive, while complete eradication, in well-established infestations, is very difficult to accomplish [19]. Management is possible however [19], and insect-based biological control is considered the best long-term option [20]. One of the candidate agents [21] is *Lasioptera donacis* Coutin & Faivre-Amiot, 1981 (Diptera: Cecidomyiidae), a monophagous (highly host-specific) midge which completes its life-cycle only on *A. donax* [20] and whose larvae interfere with the giant reed’s leaf development, compromising its photosynthetic function. The damage caused by the larvae of *L. donacis* to the leaves of *A. donax* decreases the density of the foliage produced by the reeds, resulting in greater light penetration and potentially a revival of the native plant community in areas invaded by *A. donax* [22]. Larval infestation is always associated with the presence of a saprophytic fungus, *Arthrinium arundinis* Corda (Xylariales: Apiosporaceae), which is believed to provide a trophic resource for the larvae in the reed leaf [17,21,23]. Indeed, *L. donacis*, like other midges of the genus *Lasioptera* Meigen, 1818, is believed to be sapromycophagous, feeding on the fungus which grows in the leaf sheath galleries in a symbiotic association with reed and larvae [24].

The larvae also interacts with a parasitic nematode, *Tripius gyraloura,* Poinar and Thomas, 2014 (Aphelenchoidea: Sphaerulariidae), which can be considered the natural enemy of *L. donacis* [25]. The adult female of the nematode penetrates the integument of the midge larvae and, once the insects have matured into adults, it attacks their ovaries, replacing the midge’s eggs with its own larvae and causing the sterilisation of infected female midges [25].

In spite of our knowledge of these complex interactions, it has not yet been demonstrated whether the larvae of the leaf-mining midge depend on mycelia or are able to feed directly on the reed, especially during the reed’s early growth stages, when its nutritional content and palatability might be higher [18,26]. Furthermore, although the effects of the nematode on the fecundity of gall midge adults are known [25], whether the nematode interferes with gall midge larval feeding behaviour, thereby reducing the damage that they cause to the leaves of the plant, has not yet been evaluated. This question is crucial and needs to be settled before the release of *L. donacis* as a biological control agent of *A. donax*. Therefore, the goals of this study were to (i) deepen our knowledge of the plant–fungus–insect trophic interactions and (ii) understand the role of the nematode in the feeding behaviour of the midge larvae.

In the present study, the trophic relationships between the reed, the fungus, and the larvae of the gall midge, together with the possible influence of the parasitic nematode on larval diet, were evaluated by means of stable isotope analysis (SIA). SIA is especially useful because it makes it possible to understand the trophic relationships between organisms. It can thus be used to develop models of food webs [27,28,29,30] and to define trophic niches and feeding preferences [27,31,32]. In addition, SIA has also proven to be suitable for studies of pest control and biological invasions [33,34]. The two elements most commonly employed in these studies are nitrogen (N), which exhibits stepwise enrichment with trophic transfer, and carbon (C), used to determine the original sources of dietary carbon [27,29,30]. The isotopic values of these elements in consumer tissues reflect those of the food sources in a predictable manner, since tissues are synthetised from the nutrients assimilated and ingested [27,28,29,30]. In this context, SIA is considered a suitable technique for quantifying the assimilated larval diet of *L. donacis*, not easily measurable by other techniques such as direct observation or analysis of stomach contents. To determine the proportional consumption of reed and fungus by the gall midge larvae, Bayesian stable isotope mixing models were used to identify the proportional contributions of single resource pools to a larval diet [35]. Furthermore, in order to evaluate the effects of habitat and associated reed quality on the relationships between the species, samples were collected from a number of sites and the isotopic signatures and diets of midges were compared. Since high C:N ratios, associated with low nitrogen content, could reduce the palatability of the plants for primary consumers [18,26], the effect of the reed’s C:N ratio on the feeding behaviour of midge larvae was also determined. As for *A. donax* specifically, C:N ratios greater than 17 are known to make mature leaves unattractive to herbivores, which may induce them to shift their diet toward younger and N-richer leaves, or to other food sources [18,26]. Changes in the attractiveness of food sources can in turn affect the relationships between herbivores and their natural enemies [36].

## 2. Materials and Methods

### 2.1. Lasioptera donacis Life Cycle

*Lasioptera donacis* belongs to the Cecidomyiidae family, which includes a large number of gall-forming species. Most of these species are strictly monophagous, completing their life-cycle on one plant host [37]. The genus *Lasioptera,* one of the largest of the Cecidomyiidae family, comprising 129 species [38], is defined taxonomically by the presence of mycangia. The function of these structures, in the postabdomen and in the ovipositor of adult females, is the transport of fungal conidia. Larvae of the genus *Lasioptera* are symbiotic with the fungus they feed on [39,40]. *Lasioptera donacis* is a monophagous species and the only host plant in which it manages to complete its life cycle is *A. donax* [20]. It is believed to establish a symbiotic relationship with the saprophytic fungus *A. arundinis* whose conidia are accidentally transported by adult females [20,21,23]. After mating, adult females lay eggs in several clutches of 15–25 each, inside pre-existing holes or under the leaf sheath surrounding the main stem [20]. Fungal conidia are probably deposited by the females during oviposition through the mycangia [20]. The saprophytic fungus develops soon after the eggs are laid, while the eggs hatch after about 10–12 days. The three larval stages of the gall midge develop entirely within the reed foliar sheath, surrounded by the saprophytic fungus, which provides a trophic substrate for the larvae [21]. One month after oviposition, they pupate and the newly emerged adults (5 mm) mate immediately. The females lay a new generation of eggs on leaf sheaths of the same reed from which they emerged or on a neighbouring one. At the beginning of the breeding season (in Central Italy from the end of April to mid-May), newly emerged females start to oviposit on the internodes close to the ground of new green shoots. Only later in the season, when the reed is well developed, is it possible to record oviposition on more apical internodes. A number of females can successively lay on the same leaf sheath, leading to overlapping generations of larvae [20,21,22].

### 2.2. Sampling and Stable Isotope Analysis

Samples of *A. donax, A. arundinis* and *L. donacis* were collected in September 2017 from six sites (A, B, C, D, E and F) close to small waterways or pools near Rome (Italy), in areas differing in type (urban, agricultural and open countryside) but having similar exposure to light and wind and similar conditions in terms of temperature and relative humidity (Table S1).

**Figure 1:**
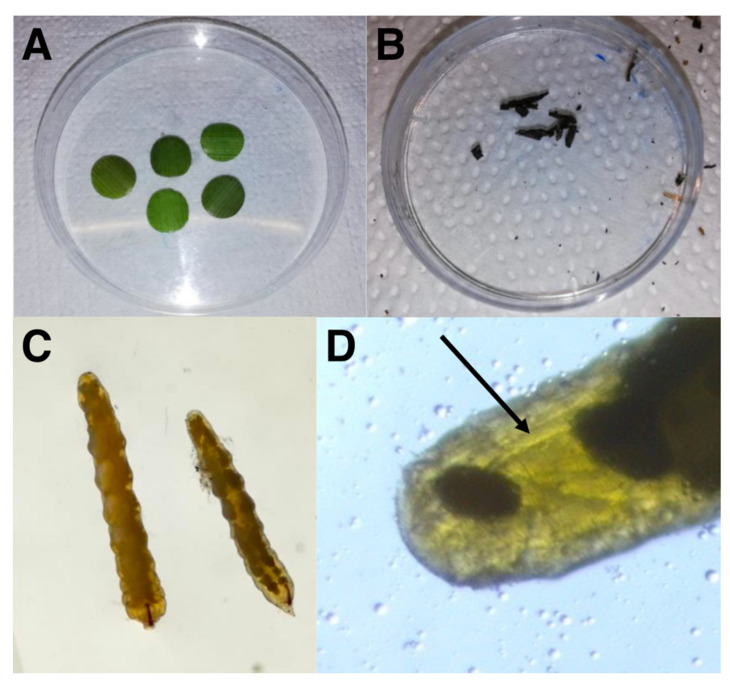
(**A**) Leaf-discs of *Arundo donax*; (**B**) mycelium of *Arthrinium arundinis* extracted from leaf sheath mesophyll; (**C**) optical microscope image of *Lasioptera donacis* larvae; (**D**) optical microscope detail of *L. donacis* abdomen parasitised by *Tripius gyraloura*. The arrow indicates the parasitic nematodes.

At each sampling site, eighteen reeds were randomly collected, stored in refrigerated boxes and transported to the laboratory. In the laboratory, portions of the leaf sheath (both healthy and colonised by the fungus), the mycelium of the fungus and the gall midge larvae were collected from the second to the sixth internode of *A. donax*, where infestation was high. No differences were observed between leaf sheaths in the number midge larvae present in the mesophyll, and therefore one internode leaf sheath per reed was randomly analysed. Specifically, the leaf sheath of the reeds, healthy or infected by the fungus, was isolated using a 1 cm Ø hollow punch. The fungal biomass and larvae were gently extracted using a stereomicroscope. Given that nematodes inside the mature larvae can be visually detected at the end of their abdomen under a stereomicroscope (Figure 1), the larvae were divided into two groups, parasitised and not parasitised by the nematode.

In order to determine C and N stable isotope values and relative C and N content, all samples were stored at −80 °C, freeze dried for 24 h and ground to a fine homogenous powder [30] using a ball mill (Fritsch Mini-Mill Pulverisette 23). Powder samples were weighed (2.5 ± 0.2 mg for plant and fungal samples and 0.5 ± 0.1 mg for nematodes and insect larvae) and inserted into tin capsules. Where possible, larvae were weighed individually (otherwise they were pooled to reach the minimum necessary weight), after which they were combusted in an Elementar vario MICRO cube analyser coupled with an Isoprime 100 mass spectrometer operating as a continuous flow system [41]. N stable isotope ratios (^15^N:^14^N) and C stable isotope ratios (^13^C:^12^C) were expressed in δ (‰) units in accordance with the following equations: δR (‰) = [(Rsample − Rstandard)/Rstandard] * 10^3^, where R is the heavy-to light isotope ratio of the element [42]. Results were calibrated with reference to International Atomic Energy Agency reference materials (IAEA-CH-3, IAEA-CH3 and USGS24 for δ^13^C; IAEA-N1, IAEA-N2 and USGS25 for δ^15^N). Measurement errors associated with the linearity and stability of the mass spectrometer were typically smaller than 0.05‰, while the standard deviation of repeated measurements of the calibrated internal standard material (IAEA-600 Caffeine, one replicate every 10 analyses) was typically ± 0.02‰ for δ^13^C and ± 0.07‰ for δ^15^N.

### 2.3. Data Analysis

Analysis of variance (one-way ANOVA) and Tukey’s HSD post-hoc test were performed to check for (a) differences between sites in the isotopic signatures of reeds, fungus and larvae, and in the C:N ratio of reeds, and (b) differences in isotopic signatures between reeds, fungus and larvae at all sites. The proportional contribution of reed and fungus to the diet of *L. donacis* larvae was estimated using Bayesian stable isotope mixing models (SIMMs) [37]. SIMMs calculate estimated dietary proportions with multiple error structures and the incorporation of fixed and random effects [35]. SIMMs require three inputs: (i) the isotopic values of the consumers (i.e., isotopic values of *L. donacis* larvae), (ii) the isotopic values of potential sources (i.e., isotopic values of reed and fungus, potential food sources for larvae) and (iii) the trophic enrichment factors (TEFs, i.e., the differences in isotopic values between a consumer and its resource due to the isotopic fractionation that occurs during metabolic processes). To compare the diets of larvae between sites, we ran a model with the isotopic signatures of larvae as mixtures and the isotopic signatures of reeds and fungus (separated for each site) as sources. The model structure had “Site” as a fixed effect and “Larvae ID” as a nested random effect. To compare the diets of larvae infected and not infected with nematodes, we also ran a SIMM with “Site” and “Infection status” as fixed effects. The TEFs used were Δ^15^N = 2.1 ± 0.2‰ and Δ^13^C = 0.3 ± 0.14‰ [43]. It was not possible to refer to any previous work using stable isotopes to characterise the diet of *L. donacis*. For all SIMMs, we ran three Markov Chain Monte Carlo chains of 300,000 iterations each with a burn-in of 200,000 and a thinning rate of 100 iterations. Each model was checked for chain convergence by visual inspection of trace-plots and application of the Gelman-Rubin and Geweke diagnostic tests. The outputs of the models were reported as means of estimated dietary proportions with their associated 95%, 75% and 50% credible intervals (CI).

In this study, all SIMMs were run using the MixSIAR R package [35]. Finally, to test the effect of parasitism on larvae, a series of paired data *t*-tests were performed: groups of parasitised and healthy larvae were compared, in order to verify the differences in δ^15^N, δ^13^C, and the proportion of the diet accounted for by *A. donax* leaves.

To verify the effect of water deficit on the leaf sheath, the leaf δ^13^C values were correlated with the distance of the reed from water canals and pools (Pearson correlation test). Furthermore, the effects of water proximity and those of internodes (from 2 to 6) on leaf C:N ratios were tested (Pearson correlation test).

## 3. Results

### 3.1. Isotopic Signatures of Reeds, Fungus and Gall Midge Larvae

Small but significant differences in mean δ^13^C values were observed between reeds, fungus and larvae (Table 1). However, in pairwise comparisons the differences were statistically significant only between reeds and larvae and not between reeds and fungus or between fungus and larvae. The average isotopic C difference (Δ^13^C‰) between larvae and reeds was 1.2 ‰ (Table 1).

Differences also existed between mean δ^15^N values (Table 1). Specifically, the δ^15^N values of reeds were significantly lower than those of fungus and larvae, which did not differ statistically from each other. The average isotopic N difference (Δ^15^N‰) between larvae and the reeds was 3.4‰ (Table 1).

**Figure 2:**
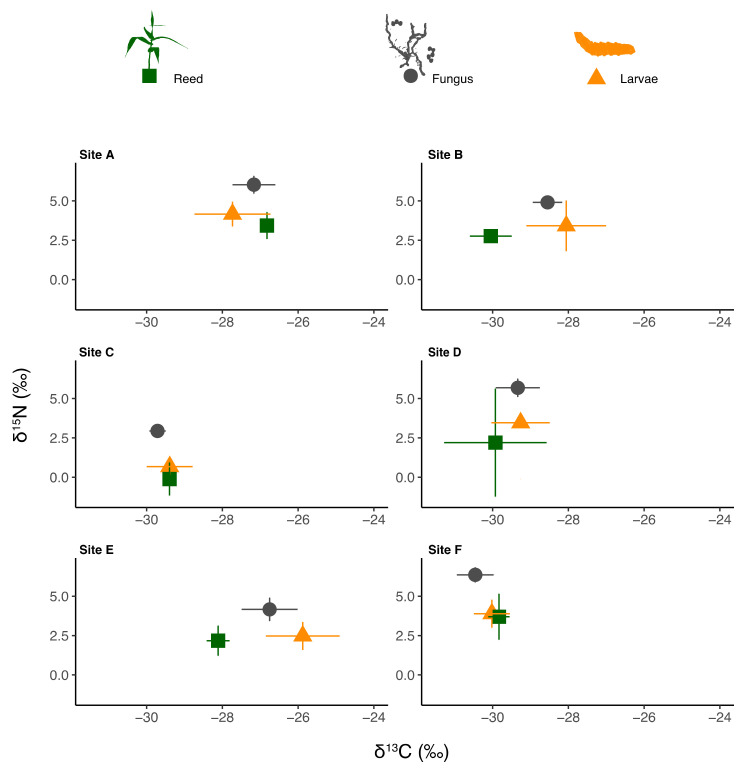
Isotopic biplots of the average δ^15^N (‰) and δ^13^C (‰) values of *A. donax* (green squares), *A. arundinis* (grey dots), and *L. donacis* (orange triangles) at sampling sites. Error bars represent standard deviation. The trophic enrichment factors (TEF) were subtracted from the isotopic values of *L. donacis*.

Differences were found between sites in the δ^13^C values of reeds, fungus and larvae (Figure 2; one-way ANOVA: F_5,13_ = 13.97, F_5,13_ = 25.91, and F_5,125_ = 58.36, respectively, always *p* < 0.001).

These differences were not correlated with the distance from canals or pools (Pearson correlation test, n.s.). The δ^15^N values of the reeds did not differ statistically between sites, although they were very low at site C, and slightly higher than the remaining ones at site F (Figure 2; one-way ANOVA, F_5,13_ = 2.15, n.s.). In contrast, the differences between sites in the δ^15^N values of both fungus and larvae were statistically significant (one-way ANOVA; F_5,13_ = 3.23, and F_5,125_ = 5.26, respectively, *p* < 0.05).

### 3.2. Diet of Gall Midge Larvae: Influence of Reed Quality and Parasitism by T. gyraloura

The larvae consumed the reeds and the fungus in varying percentages, thus displaying a variable diet. On average, fungus accounted for 65% of the diet of the midge larvae. The highest consumption of reeds was observed at site F and the lowest at site B and vice versa for the fungus (Table 2).

**Figure 3:**
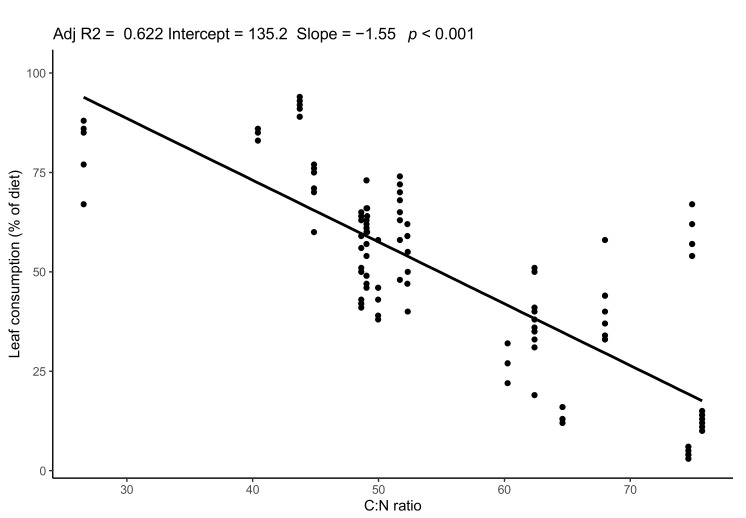
Correlation between the proportion of consumption by gall midge larvae and the C:N ratio of the reed. Each dot represents the estimated proportion of an individual larva.

Variability in the reed carbon-nitrogen ratio (C:N ratio) was observed between samples but not between sites (one-way ANOVA, F = 2.1, df = 17, n.s.). The C:N ratio was not correlated with distance from water bodies nor with the internodes (Pearson correlation test, n.s.). The consumption of reeds as a percentage of the gall midge larval diet also varied within sites and was inversely correlated with the C:N ratio (Figure 3).

The overall low nitrogen content in the leaf sheaths was associated with a rather high C:N ratio, on average equal to 55.0 ± 13.2%, which led to lower average consumption of reeds than fungus.

**Figure 4:**
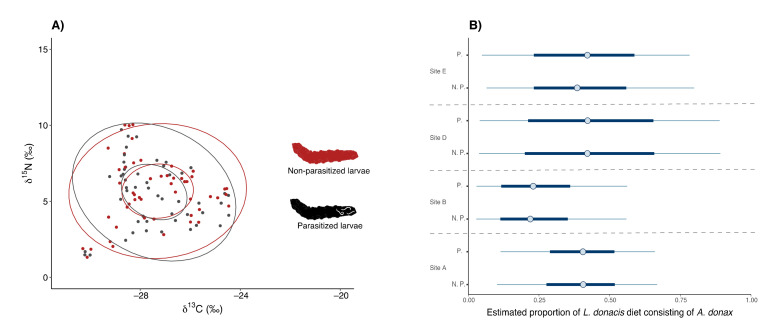
(**A**) Isotopic biplot of non-parasitised (N.P., red dots, red ellipses) and parasitised (P., blue dots, blue ellipses) larvae. Inner ellipses represent 40% of the samples, while outer ellipses represent 95%. (**B**) Estimated proportions of consumption accounted for by reeds among parasitised (P.) and non-parasitised (N.P.) larvae at sites A, B, D and E. Results are reported as modal values (blue circles) with their associated 50% (thick blue lines) and 95% (thin blue lines) credibile intervals.

Parasitised larvae were found in four out of the six sites (sites A, B, D and E) in similar proportions (about 30% of the total number of larvae). No differences in carbon and nitrogen isotope signatures were observed between parasitised and non-parasitised larvae (t_at least_ = 0.09, n.s.), except for nitrogen at site E (t = 2.61, *p* < 0.05, Table S2). Parasitised and non-parasitised larvae displayed similar isotopic niches (Figure 4A) and similar percentages of reed in their diet (Figure 4B; t = 0.26, n.s.).

## 4. Discussion

The results of this SIA-based study show that: (i) midge larvae consume reeds and the fungus in variable proportions depending on reed quality, measured as the C:N ratio [18,26]: as this falls, the proportion of the diet composed of fungus increases; (ii) the feeding behaviour of the midge larvae is not affected by parasitic nematodes. From a methodological point of view, stable isotope and elemental analyses and Bayesian mixing models enabled us to obtain valuable and precise information regarding the trophic relationships, not yet completely clarified, of *L. donacis*. Indeed, this approach is very useful in disentangling trophic interactions, especially when dealing with very small organisms such as invertebrates or cases where consumers live immersed in a mixture of resources (as in the case of *L. donacis*) and classical methods (i.e., direct observation and/or stomach content analyses) are very difficult to apply. On the practical side, knowledge of these trophic interactions is key to understanding their ecology and assessing the adequacy of biological control measures, and it is also useful for management planning. The arundo gall midge is considered the third most promising agent, after with the stem-galling wasp *Tetramesa romana* Walker (Hymenoptera: Eurytomidae) and the rhizome-feeding armoured scale *Rhizaspidiotus donacis* (Leonardi, 1920) (Homoptera: Diaspidae), for the biological control of *A. donax*. Its release is permitted in North America and Mexico [20,22]. *L. donacis* is highly host-specific and its monophagy to *A. donax* poses no significant threats to other native or economically valuable plants, despite partial larval development being observed in *Phragmites australis* (Cav.) Trin. Ex Steud [20]. Some of the midge’s larval stages develop entirely in the mesophyll of the stem leaf sheaths of the reed, where they have been seen feeding together in groups or clusters. The development of the first larval stages generally occurs before the accumulation of fungal biomass [20,21]. According to Rohfritch [40] in this symbiotic relationship, the fungus can provide, in exchange for being transported to new hosts, initial nourishment for the larvae of *Lasioptera* spp. and protection from external agents. Midge larvae were once believed to feed on the fungus and not directly on the reed. Based on the presence of rudimentary mouthparts, the enlargement of the salivary glands and the lack of frass accumulations, Thomas and Goolsby [21] argue that midge larvae have a liquid diet as a result of the extraoral digestion of fungal material [21]. However, from the isotopic biplots it was observed that the larvae were positioned both above and between the reed and the fungus, indicating that they feed on both resources. It could thus be described as an omnivore (i.e., an organism that feeds on more than one trophic level). *Arundo donax* is the only trophic source of the saprophytic fungus [20], and for this reason the carbon isotopic values of the fungus and the reed were very similar. However, the average isotopic differences between the larvae and the reed (Δ^13^C = 1.2. ‰ and Δ^15^N = 3.4 ‰) placed the larvae, with a certain degree of variability between sites, on the second trophic level, i.e., only one level above the reed. This result leads us to reject the hypothesis that larvae feed only on the fungus [17,21,23]. Indeed, both δ^13^C and δ^15^N clearly indicate that midge larvae also feed directly on the leaf sheath of the giant reed. The outputs of the Bayesian mixing models suggested that the proportions of the larval diet attributable to reeds and fungus vary between sampling sites and sometimes even within sites. This is due to variation in the quality of the reed leaf sheath as a food resource, measured as the inverse of the carbon-nitrogen ratio (C:N). Specifically, the lower the nitrogen content of the reed, the higher the C:N ratio, and the lower the direct consumption of this food source by the gall midge larvae. Indeed, the overall low nitrogen content of reed leaf sheaths was reflected by an average C:N ratio of 55 (Table 1), substantially beyond the ratio of 17 at which the leaves become unattractive to phytophagous consumers [18,26]. This led to reduced direct consumption of reeds by the larvae, which shifted their diet to saprophytes, which are an N-rich and highly energetic food source [18,26].

The C:N ratio of plants reflects the protein content, which is considered one of the most important nutritional characteristics of a food source [44,45,46]. Proteins account for about 7% by mass of giant reed leaf fibres and 6% of leaf parenchyma, higher than the internal stem and other parts of the plant [47,48]. The synthesis of most proteins depends on the amount of nitrogen that the plant absorbs, which in turn depends on the input of nitrogen to the soil [26,49,50]. A certain variation in the nitrogen isotopic signatures of reeds between locations could depend on the source of the nitrogen, since it can originate from a variety of natural and anthropogenic processes and sources, such as wastewater or synthetic inorganic fertilisers [51,52,53]. The significant differences observed in the carbon isotopic signatures were not due to water deficit, related to the distance from water canals and pools, as observed in other studies [54]. Although no statistical differences were found in leaf quality (as C:N) from internodes 2 to 6, this topic merits further investigation. Leaf quality is relevant to the biological control of *A. donax*, since both *L. donacis* and its associated saprophyte complete their development only on the giant reed [20], and changes in reed leaf sheath quality could affect the feeding choices of the gall midge. *L. donacis* could include parts of the reed in its diet to compensate for the mismatch between the development of the first larvae and that of the trophic substrate providing good quality energy. Therefore, the gall midge could promote control of *A. donax* both directly, by feeding on the leaves, and indirectly, by promoting the introduction and spread of the fungal substrate and, thus, the degradation of the giant reed [49]. According to Goolsby and colleagues [20], the release of *L. donacis* could complement the control activity of the other two agents, the arundo wasp and arundo scale, and enhance the damage and stress inflicted on *A. donax*.

While soil quality and N-input can have a role in reed quality and thus in the feeding behaviour of midge larvae, our results showed that their infection by nematodes does not affect the proportional consumption of leaf sheath and fungal mycelium. This is to be expected given that female nematodes become aggressive toward the host only during its pupal or adult phase, when their offspring in the ovaries of the midge replace the insect’s eggs [25]. During the larval phase, the female nematodes inside the midge larvae merely absorb nutrients via the skin [21,25]. Furthermore, the results indicated that there were no significant differences between the groups of parasitised and non-parasitised larvae in terms of the isotopic signatures of either nitrogen (δ^15^N) or carbon (δ^13^C), suggesting that *T. gyraloura* is extremely well-adapted. Its life cycle is fully correlated with that of the gall midge, and the fact that its presence does not modify the larval feeding behaviour suggests evolution by the parasite towards low virulence to the host insect [55,56,57]. However, as observed for the two other *Tripius* species, * T. gibbosus* (Leuckart) and *T. sciarae* (Bovien), and their respective hosts, *T. gyraloura* can be considered a natural enemy of *L. donacis* [25]. Indeed, the presence of the nematode causes sterilisation of the adult insect and could thus reduce its fitness and effectiveness as a biological control agent. Therefore, as suggested by Poinar and Thomas [25], its eradication from laboratory colonies and the development of parasite-free populations of gall midge should be considered before their release into the wild.

## 5. Conclusions

This study highlighted for the first time the occurrence of feeding interaction between the reed and the gall midge larvae. *L. donacis* turns out to be an ‘omnivore’, feeding on both reed and fungus in variable proportions, and not exclusively a third-level consumer that consumes fungus growing on reeds. This indicates a closer trophic relationship between the midge and the target weed than what has been described in previous studies [21,23]. Moreover, several records highlight the close relationship between gall midge species belonging to the Cecidomyiidae family and their target host plant species [37,58]. The results of this study therefore lead to refute the established notion that gall midges are strictly fungus feeders. In this study, stable isotope analysis and Bayesian Mixing models illustrated how midge larvae can optimise their foraging based on the trophic quality of the reed, thus maximising net energy intake [59] regardless of the abundance of the fungus. The models also suggested that the infecting nematode does not impact the feeding behaviour of the larvae, although it may influence their fitness.

The study of this multi-trophic system can lay the foundations for studies of the ecological aspects of *A. donax* and the food chains of other potential control agents for this plant that have not yet been investigated. Stable isotope analysis has proved to be a useful tool in the biological control of invasive alien plants, especially for identifying and understanding the feeding physiology and behaviour of many phytophagous arthropods assessed as potential biocontrol agents. *Lasioptera donacis* appears to be an effective control agent of reeds, especially when their nitrogen content is high, as in plants growing in nutrient-enriched soils or wetlands [60]. However, the trophic plasticity of the larvae enables them to survive even in less favourable N conditions by relying for their diet on fungus. This needs to be taken into account when implementing biological control measures, since in food webs with three trophic levels the attractiveness of the basal resources is known to directly and indirectly affect the presence and movement of the associated community of arthropods, including the potential natural enemies of pests [26,36].

## Figures and Tables

**Table 1:** Mean carbon and nitrogen stable isotope values and C:N ratios of reeds, fungus and midge larvae. For each parameter, different superscript letters (a, b, c) indicate statistical differences between reeds, fungus and larvae (one-way ANOVA and Tukey post-hoc comparisons; *p* < 0.05).

	δ^13^C (‰)	δ^15^N (‰)	C:N	Sample Size
	Mean ± S.D.	Mean ± S.D.	Mean ± S.D.	N°
*A. donax*	−29.0 ± 1.3 ^a^	2.5 ± 1.9 ^a^	55.0 ± 13.2 ^a^	18
*A. arundinis*	−28.7 ± 1.5 ^a,b^	5.1 ± 1.7 ^b^	22.1 ± 4.4 ^b^	18
*L. donacis*	−27.8 ± 1.6 ^b^	5.4 ± 2.0 ^b^	10.9 ± 1.9 ^c^	124

**Table 2:** Diet of *L. donacis* based on stable isotope mixing model outputs. Values are reported as mean probabilities and upper and lower 95% credibile intervals.

Site	Reed Consumption			Fungus Consumption			N° *L. donacis*
	Mean (%)	2.5%	97.5%	Mean (%)	2.5%	97.5%	
A	32.1	10.7	57.1	67.9	42.9	89.3	27
B	6.8	0.8	20.4	93.2	79.6	99.2	26
C	58.4	19.5	82.9	41.6	17.1	80.5	9
D	17.8	1.5	57.4	82.2	42.6	98.5	24
E	8.8	0.9	25.0	91.2	75.0	99.1	25
F	85.9	72.6	96.4	14.1	3.6	27.4	13

## Data Availability

Not applicable.

## Supplementary Materials

The following supporting information can be downloaded at: https://www.mdpi.com/article/10.3390/biology11121805/s1, Table S1: Sampling sites. For each site geographical coordinates and description of the area are reported; Table S2: C and N isotopic signatures of *L. donacis* with and without parasites in four sampling sites. The *t*-test refers to the comparison between parasitized and non-parasitized larvae in the same site.
