# Supplementary material for: The Feeding Behaviour of Gall Midge Larvae and Its Implications for Biocontrol of the Giant Reed: Insights from Stable Isotope Analysis
Source: Biology (Basel). 2022 Dec 12;11(12):1805. doi: 10.3390/biology11121805 (PMC9775122; doi:10.3390/biology11121805)
Supplement: Supplementary file 1 [file biology-11-01805-s001.zip › biology-2036254-supplementary.pdf]

The feeding behaviour of gall midge larvae and its implications for biocontrol of the giant reed: insights from stable isotope analysis

Giulio Careddu[1,2], Marcovalerio Botti[1,3], Massimo Cristofaro[3], Simona Sporta Caputi[1,2,*], Edoardo Calizza[1,2], Loreto Rossi[1,2] and Maria Letizia Costantini[1,]

[1] Department of Environmental Biology, Sapienza University of Rome, 00185, Rome, Italy
[2] CoNISMa, National Inter-University Consortium for Marine Sciences, 00196, Rome, Italy
[3] Biotechnology and Biological Control Agency (BBCA), 00123, Rome, Italy
[*] Correspondence: simona.sportacaputi@uniroma1.it

Supplementary material

Table S1. Sampling sites. For each site geographical coordinates and description of the area are reported.

Site	Latitude (N)	Longitude (E)	Site description
**A**	41°59′3″	12°5′25″	Next to a canal surrounded by trees that shade the site and protect it from the wind in town Cerveteri.
**B**	42°1′35″	12°15′53″	On the edge of a busy road, covered by a few pine trees, in the small village of Osteria Nuova.
**C**	41°40′44″	12°44′19″	Near a drainage channel in town of Velletri.
**D**	42°0′57″	12°22′23″	Grassy area surrounded by urban buildings in La Storta, a district in the northern part of Rome
**E**	42°8′53″	12°16′22″	On a dirt road with little traffic and vegetation, in Trevignano Romano, near Lake Bracciano.
**F**	41°33′0″	12°43′46″	Near an agricultural drainage channel next to a busy tree-lined road of village of Campo Verde.

Table S2. C and N isotopic signatures of *L. donacis* with and without parasites in four sampling sites. The t-test refers to the comparison between parasitized and non-parasitized larvae in the same site.

**Site**	**Larvae with parasites**			**Larvae without parasites**			**t-test**	
	**$\delta^{15}N$ (‰)**	**$\delta^{13}C$ (‰)**	**Sample size**	**$\delta^{15}N$ (‰)**	**$\delta^{13}C$ (‰)**	**Sample size**	**$\delta^{15}N$**	**$\delta^{13}C$**
	**Mean ± S.D.**	**Mean ± S.D.**	**N°**	**Mean ± S.D.**	**Mean ± S.D.**	**N°**		
**A**	6.5 ± 0.7	-27.7 ± 1.0	13	6.0 ± 0.8	-27.2 ± 0.9	14	n.s.	n.s.
**B**	5.5 ± 1.2	-27.5 ± 2.1	13	5.5 ± 1.8	-28.0 ± 0.9	13	n.s.	n.s.
**D**	5.5 ± 3.5	-28.9 ± 0.8	12	5.7 ± 3.8	-29.1 ± 0.7	12	n.s.	n.s.
**E**	4.2 ± 0.7	-25.8 ± 1.2	12	5.0 ± 0.9	-25.4 ± 0.7	12	**$p < 0.05$**	n.s.
